# Re-emergence of Rift Valley fever: an update and preventive measures

**DOI:** 10.1097/JS9.0000000000000132

**Published:** 2023-01-27

**Authors:** Hitesh Chopra, Kuldeep Dhama, Talha B. Emran

**Affiliations:** a Chitkara College of Pharmacy, Chitkara University, Rajpura, Punjab; b Division of Pathology, ICAR-Indian Veterinary Research Institute, Bareilly, Izatnagar, Uttar Pradesh, India; c Department of Pharmacy, BGC Trust University Bangladesh, Chittagong, Bangladesh; d Department of Pharmacy, Faculty of Allied Health Sciences, Daffodil International University, Dhaka, Bangladesh

Highlights

- Rift Valley fever (RVF) in African subcontinent country known as Mauritania.
- RVF reported to spread from domesticated animals such as cattle, buffalo, etc.
- *Aedes* species of mosquitoes, can pick the virus transmission.
- RVF virus can be spread by any of a number of different mosquito species.

WHO on dated October 20, 2022, declared the outbreak of Rift Valley fever (RVF), in African subcontinent country known as Mauritania. In Mauritania, between August 30, 2022 and October 17, 2022, about 47 cases were reported out of them most people are animal breeders and 23 deaths were reported (Fig. 1) 1. The RVF has been earlier reported to spread from domesticated animal such as cattle, buffalo, sheep, and goats. Also, the mosquito bites from infected animals can act as source of such viral outbreak. The *Aedes* species of mosquitoes, can pick the virus transmission via sucking of blood from infected animal and also the female mosquitoes can pass this virus through her eggs, which can be the new generation of infected mosquitoes. In laboratory, the airborne based viral inhalation (aerosol transmission) has been performed and but human to human transmission was not detected based on it. The RVF virus maintains the vertical transmission in *Aedes* mosquitoes at key foci regions by transfer between vector and hosts. The illness quotient can be amplified with the local mosquitoes such as *Culex*, *Mansonia*, and *Anopheles* that can act as mechanical vectors during the outbreaks. The mosquitoes present near to the irrigation system are extremely possible cause of secondary outbreak. In the year 1910, it was first detected in Kenya Rift valley, but later it was also reported in West Africa and Madagascar. In the year 2000, Saudi Arabia reported similar viral outbreak 2.

**Figure 1:**
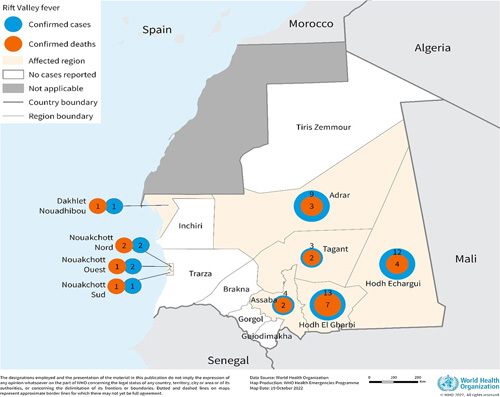
Geographical distribution of confirmed human cases of Rift Valley fever (*n*=47) and deaths (*n*=23) from nine affected wilayas in Mauritania, 30 August–October 17, 2022. *Source*: WHO 1.

The RVF virus can be spread by any of a number of different mosquito species. Different species can play various roles in maintaining the virus’s transmission, and different locations have different dominant vector species. If symptoms do manifest, RVFV has an incubation period of 2–6 days after viral introduction and can result in a number of distinct illness syndromes. RVF patients typically present with either no symptoms or a moderate illness during the time of illness that includes fever, weakness, back pain, and dizziness. Usually, patients feel better 2 days to a week after their symptoms first appear. The likelihood of coming into contact with mosquitoes and other insect vectors may increase if you spend time in rural areas or sleep outside at night in places where RVF outbreaks are common. An elevated risk of infection exists for those who butcher or handle raw meat from possibly infected animals in RVF-endemic regions. When visiting RVF-endemic regions during times of sporadic cases or outbreaks, international tourists increase their risk of infection to the virus. The majority of human diseases are brought on by direct or indirect contact with an infected animal’s blood or organs. When touching animal tissue during slaughter or butchery, helping with animal births, performing veterinary treatments, or disposing of corpses or fetuses, the virus can be transferred to humans. As a result, some occupational groups, including farmers, herders, those who work in slaughterhouses, and veterinarians, are more susceptible to infection. RVF has no FDA-approved therapies. The majority of RVF instances are minor and self-limiting, hence there is not a well-established RVF therapy. Body pains and fever are common minor disease symptoms that can be treated with common over-the-counter drugs. The majority of the time, patients recover from their illnesses 2 days to 1 week after they first appear 2. More severe instances often just require supportive treatment and may necessitate hospitalization. There are still many issues and difficulties with the management and prevention of RVF. Researchers are still looking into the risk factors for severe instances of RVF in humans as well as how the virus is maintained and disseminated across various mosquito species. To better understand how the RVF virus infection spreads and to develop practical preventive strategies, surveillance (close monitoring for RVF infection in animal and human populations) is crucial.

To anticipate and manage future RVF outbreaks, case surveillance and environmental monitoring systems may be established 3. There are several vaccinations that may be used on animals. Because numerous doses are required, inactivated, or destroyed, vaccines are impractical for routine animal field immunization. One of the first and most popular vaccinations for preventing RVF in Africa is a modified live vaccine known as the Smithburn vaccine. Although this vaccine only needs one dosage, it is known to induce birth abnormalities and miscarriages in pregnant animals and may only offer a minimal level of protection for cattle against RVF infection. There are several potential vaccines being created and tested. In laboratory tests on domesticated animals, the live-attenuated vaccination MP-12 has produced encouraging results; nevertheless, further studies are required before the vaccine may be used in the field. Recent registration and usage of the live-attenuated clone 13 vaccine in South Africa. The creation of substitute vaccines utilizing molecular recombinant constructs has shown encouraging results. The most efficient method of vector control is larviciding at mosquito breeding sites, provided that breeding sites can be precisely located and are constrained in terms of size and scope. However, at times of floods, the quantity and size of breeding sites are frequently too great for larviciding techniques to be effective. Forecasting helps with disease control by predicting climatic variables that are usually linked to a higher risk of outbreaks. RVF outbreaks in Saudi Arabia and Yemen are strongly correlated with times of above-average precipitation in Africa. Remote sensing satellite imagery makes it simple to assess and monitor how vegetation reacts to higher rainfall levels. These discoveries have made it possible to successfully create forecasting models and early warning systems for RVF utilizing data from weather/climate predictions and satellite pictures. These early warning devices might be used to discover animal cases at the earliest possible stage of an outbreak, allowing authorities to take preventative action before an epidemic spread. Forecasting and early RVF outbreak identification within the context of the new International Health Regulations (2005), along with a thorough evaluation of the risk of diffusion to new regions, are crucial to enable the execution of effective and timely control measures. In response to the 2016 Niger epidemic, WHO dispatched a multisectoral national fast response team that included representatives from the Center for Medical and Veterinary Research and the Ministry of Health (CERMES) 4. On August 31, 2016, the unit was sent out for a field investigation. The WHO Country Office in Niger offers technical and financial assistance for risk communication, case definition, case management, epidemic investigation, surveillance, and outbreak investigation. The World Organisation for Animal Health (OIE), the Food and Agriculture Organization of the United Nations (FAO), and the World Health Organization (WHO) are collaborating on animal and human health and giving Niger extra help for the epidemic response.

## Ethical approval

Not applicable.

## Sources of funding

None.

## Author contributions

H.C.: conceptualization, data curation, writing – original draft preparation, writing – reviewing and editing. K.D.: data curation, writing – original draft preparation, writing – reviewing and editing. T.B.E.: writing – reviewing and editing, visualization, supervision.

## Conflicts of interest disclosure

The authors declare that they have no financial conflict of interest with regard to the content of this report.

## Research registration unique identifying number (UIN)

None.

## Guarantor

Talha Bin Emran, PhD, Associate Professor, Department of Pharmacy, BGC Trust University Bangladesh, Chittagong 4381, Bangladesh. Tel: +880 303 356 193, Fax: +880 312 550 224. https://orcid.org/0000-0003-3188-2272.

## Provenance and peer review

Not commissioned, internally peer-reviewed.

## Data statement

No specific data collected for the above manuscript. The data in this correspondence article is not sensitive in nature and is accessible in the public domain. The data is therefore available and not of a confidential nature.
